# Development and validation of a machine learning-based model for predicting survival in patients with bladder neuroendocrine carcinoma: a large retrospective study

**DOI:** 10.3389/fonc.2026.1956255

**Published:** 2026-10-27

**Authors:** Shen Qi, Wenbo Dong, Zhaokun Shi, Yanshen Sun, Dongyan Zhang, Changxue Liu, Hao Li, Liangliang Hu

**Affiliations:** 1 Department of Neurology, Shandong Zaozhuang Municipal Hospital, Zaozhuang, China; 2 Department of Urology, Shandong Zaozhuang Municipal Hospital, Zaozhuang, China; 3 Department of Neurosurgery, Shandong Taian the First People’s Hospital of Dongping County, Taian, China; 4 Department of General Surgery, Zaozhuang Hospital, Shandong Guoxin Yiyang Group, Zaozhuang, China; 5 Department of Imaging, Zaozhuang Hospital, Shandong Guoxin Yiyang Group, Zaozhuang, China; 6 Department of Urology, The Affiliated Hospital of Qingdao University, Qingdao, China; 7 Department of Urology, Zaozhuang Hospital of Dongfang Hospital Beijing University of Chinese Medcine, Zaozhuang, Shandong, China

**Keywords:** bladder neuroendocrine carcinoma, machine learning, prognostic model, SEER database, survival prediction

## Abstract

**Background:**

Bladder neuroendocrine carcinoma (BNEC) is a rare yet highly aggressive malignancy with an extremely poor prognosis. The current TNM staging system provides limited prognostic information and validated prognostic prediction tools are lacking. This study aimed to develop and validate a machine learning (ML)-based model for individualised survival prediction in BNEC patients using large-scale population data.

**Methods:**

Data from pathologically confirmed BNEC patients from 2004 to 2021 were extracted from the US Surveillance, Epidemiology, and End Results (SEER) database. Univariate and multivariate Cox regression analyses were performed to identify independent prognostic factors. On the basis of the independent prognostic factors, eight machine learning models, namely, random forest (RF), gradient boosting machine (GBM), LASSO-Cox, CoxBoost, support vector machine (SVM), XGBoost, SuperPC, and plsRcox, were constructed using the training set (70% of the total sample). Model performance was comprehensively evaluated on the validation set (30% of total samples) using the C-index, time-dependent receiver operating characteristic (ROC) curves, calibration curves, and decision curve analysis (DCA).

**Results:**

A total of 2117 patients with BNEC were included in the final analysis. Multivariate analysis revealed age >74 years (HR = 1.51, p<0.001), male sex (HR = 1.17, p=0.038), advanced T stage (T2–T4), positive nodal status (N1–N2), distant metastasis (M1, HR = 1.99, p<0.001), surgery (TURBT: HR = 0.75, P = 0.017; RC: HR = 0.36, P<0.001), radiotherapy (HR = 0.79, p=0.002), and chemotherapy (HR = 0.43, p<0.001) as independent prognostic factors. Among the eight machine learning models, the GBM algorithm demonstrated the best performance in both the training and validation sets, with AUC values for 1-year, 3-year, and 5-year survival prediction being 0.846/0.737, 0.825/0.730, and 0.816/0.742, respectively. Decision curve analysis (DCA) demonstrated greater clinical net benefit across a broad range of threshold probabilities, while the calibration curves indicated high agreement between the predicted and observed outcomes. Risk stratification based on the GBM-derived risk score revealed significant differences in survival between the high-risk and low-risk groups (p < 0.0001).

**Conclusion:**

The GBM-based machine learning prognostic model demonstrated comparatively favorable performance in predicting survival outcomes among patients with BNEC. This model may provide a useful tool for individualized prognostic assessment and risk stratification, although further validation in multicentre prospective cohorts is warranted.

## 1 Introduction

Bladder neuroendocrine carcinoma (BNEC) accounts for less than 1% of all bladder malignancies but is characterized by highly aggressive biological behaviour, with a significantly poorer prognosis than urothelial carcinoma of the bladder (1–3). Owing to its low incidence, standard treatment regimens often draw from small cell lung cancer or neuroendocrine tumours at other sites and lack high-level evidence-based medical support (4–6). Currently, prognosis assessment for BNEC relies primarily on the American Joint Committee on Cancer (AJCC) TNM staging system. However, this system does not sufficiently account for numerous factors, such as patient characteristics and treatment modalities, resulting in limited predictive efficacy. In recent years, ML has demonstrated significant potential in medical prognosis prediction. ML algorithms efficiently process high-dimensional, nonlinear data and capture complex interactions between variables, thereby providing more accurate predictions than traditional statistical methods (7–9) do. Although previous studies have attempted to construct prognostic nomograms for BNEC (10, 11) the systematic application and comparison of multiple advanced ML algorithms for predicting the prognosis of BNEC remain unexplored. Therefore, based on large-scale data from the SEER database, the aims of this study are as follows: 1. Systematic screening of independent prognostic factors influencing BNEC prognosis. 2. Construction and comparison of the performance of eight mainstream machine learning survival prediction models. 3. Identification of the model with the most favorable predictive performance and comprehensive internal validation and clinical applicability assessment. This study aims to fill the research gap in BNEC prognosis prediction and provide a potential methodological framework for individualized prognostic assessment and risk stratification.

## 2 Materials and methods

### 2.1 Data sources

Data were sourced from the publicly available Surveillance, Epidemiology, and End Results (SEER) database of the National Cancer Institute. Case information from 2004 to 2021 was extracted using SEER*Stat software (version 9.0.42). The inclusion criteria were as follows: primary neuroendocrine carcinoma of the bladder (primary site codes: C67.0–C67.9); pathologically confirmed bladder neuroendocrine tumour with histological classification (ICD-O-3 codes: 8013/3 large cell neuroendocrine carcinoma, 8041/3 small cell carcinoma, 8042/3 oat cell carcinoma, 8045/3 mixed small cell carcinoma, 8240/3 carcinoid, 8246/3 neuroendocrine carcinoma, 8680/3 malignant paraganglioma, 8693/3 adrenal paraganglioma, and 8700/3 pheochromocytoma); age ≥ 18 years at diagnosis; and complete clinical information with ≥ 1 month of follow-up. The exclusion criteria included missing key clinical data (e.g., T/N/M stage), a lack of follow-up information, age < 18 years, and unknown survival times. Ultimately, 2,117 eligible patients were selected for analysis from 2,858 candidate cases (**Figure 1**). The variables included demographic characteristics (age, sex, and race), oncological features (primary site, histologic subtype, AJCC stage, and T/N/M stage), and treatment information (surgery, radiotherapy, and chemotherapy). For radiotherapy and chemotherapy, the SEER “No/Unknown” category was retained as a single category in the analysis. Continuous variables (e.g., age) were grouped on the basis of median values, and all categorical variables were converted into dummy variables before model analysis. Data for this study were sourced from the publicly available SEER database, eliminating the need for additional ethical review.

**Figure 1:**
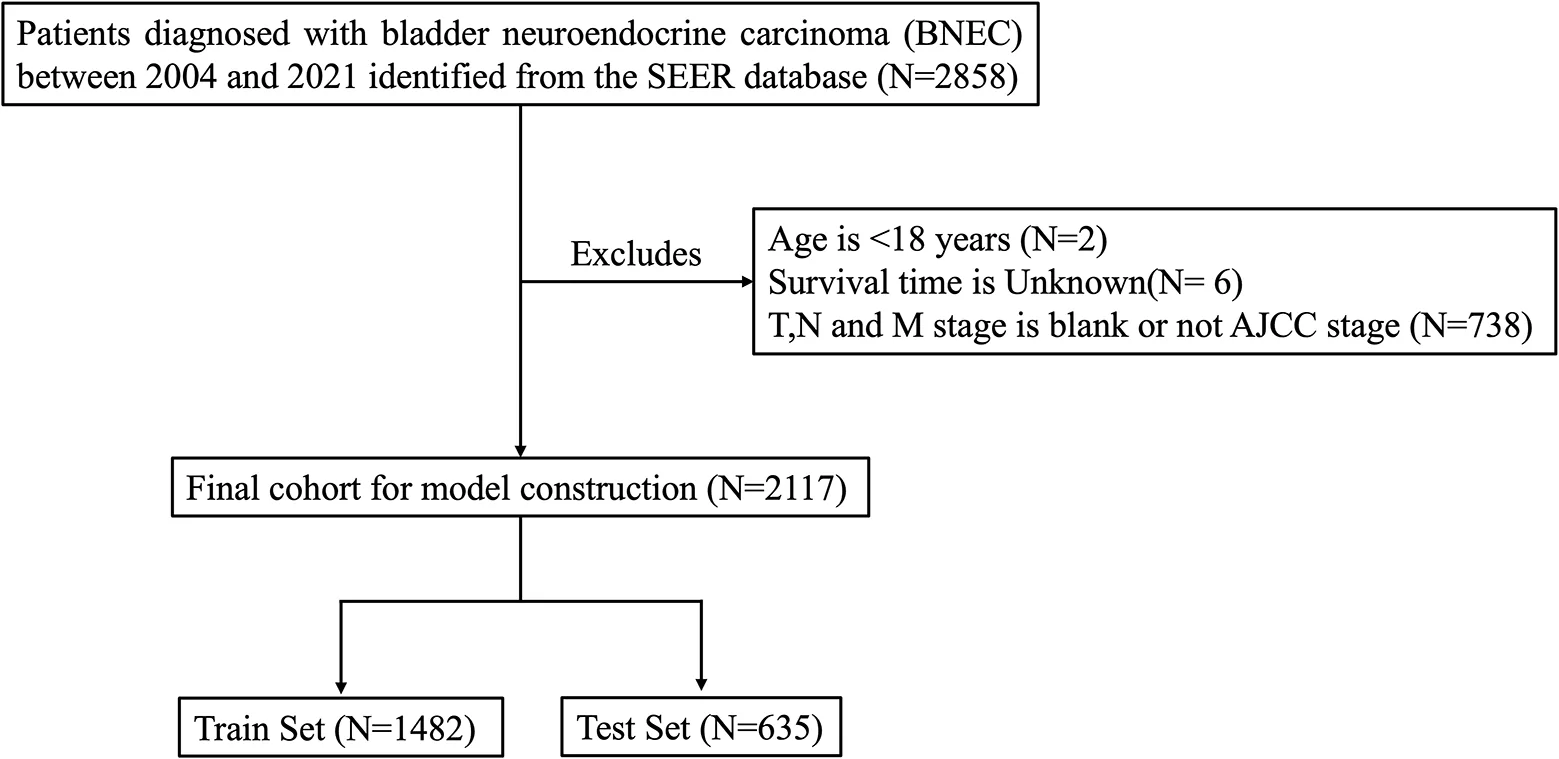
Flowchart of patient screening in the study.

### 2.2 Model training and validation

Patients were randomly assigned to a training set (70%) or a validation set (30%) using a 7:3 ratio. Univariate and multivariate Cox regression analyses were performed to identify prognostic factors associated with overall survival (OS) in patients with BNEC. Eight machine learning survival prediction models, namely, random survival forest (RSF), gradient boosting machine (GBM), LASSO-Cox, CoxBoost, SurvivalSVM, XGBoost, SuperPC, and plsRcox, were constructed using the training set. The predictive performance of each model was subsequently evaluated in the validation set. Model performance was assessed in terms of discrimination, calibration, and clinical utility. Discrimination was evaluated using the concordance index (C-index) and time-dependent receiver operating characteristic (ROC) analysis. Calibration was assessed by comparing predicted and observed survival probabilities using calibration curves. Decision curve analysis (DCA) was performed to assess the net benefit of the model across a range of threshold probabilities. Finally, patients were stratified into high-risk and low-risk groups according to the median risk score of the selected model, and Kaplan–Meier survival analysis with log-rank testing was performed to evaluate differences in survival between the two groups (12, 13).

### 2.3 Survival modelling and model performance evaluation

Overall survival (OS) was defined as the time from the date of diagnosis to death from any cause or the last available follow-up. Patients who were alive at the last follow-up were considered right censored. Survival time was recorded in months, and the event indicator was defined as 1 for death and 0 for censored observations. Eight machine learning survival models were developed, including random survival forest (RSF), gradient boosting machine (GBM), LASSO-Cox, CoxBoost, SurvivalSVM, XGBoost, supervised principal components (SuperPC), and partial least squares regression for Cox models (plsRcox). The RSF model was implemented using a log-rank splitting rule. The GBM model was fitted using a Cox proportional hazards objective. LASSO-Cox regression was performed using an L1-regularized Cox proportional hazards model, with the penalty parameter selected by 10-fold cross-validation using the 1-SE criterion; variables with non-zero coefficients at the selected penalty were subsequently entered into a multivariable Cox proportional hazards model. CoxBoost was implemented using component-wise boosting for Cox survival modelling. SurvivalSVM was fitted using a linear kernel with the specified pairwise difference construction and quadratic programming optimization. XGBoost was implemented using the Cox proportional hazard’s objective (survival:cox), with the Cox negative log-likelihood used as the evaluation metric. SuperPC was fitted for censored survival outcomes using survival time and censoring status, whereas plsRcox was implemented using partial least squares regression for Cox survival modelling.The predicted risk scores generated by each model were used for subsequent performance evaluation. The concordance index (C-index) was calculated using the pec package to assess the discriminative ability of each survival model, with right-censored observations accounted for using inverse probability of censoring weighting (IPCW). C-index values were evaluated at 5-month intervals during follow-up. Time-dependent ROC analysis was performed using the riskRegression package. For each model, the predicted risk score was incorporated into a Cox proportional hazards model and evaluated using the Score function. Time-dependent AUCs were calculated at 5-month intervals during follow-up, with right-censored observations accounted for using IPCW. Higher C-index and AUC values indicated better discriminative performance.

### 2.4 Statistical analysis

All the statistical analyses and machine learning modelling were performed using R software (version 4.2.3). Categorical variables were compared using chi-square (χ²) or Fisher’s exact tests. Cox proportional hazards regression models were employed to analyse the factors influencing overall survival (OS). In univariate analysis, hazard ratios (HRs), 95% confidence intervals (CIs), and p values were calculated, with p ≤ 0.05 as the criterion for statistical significance. Variables meeting these criteria were further included in multivariate analysis to identify independent prognostic factors. The results are expressed as hazard ratios (HRs) and 95% confidence intervals (CIs). Overall survival (OS) was estimated using the Kaplan–Meier (KM) method, and survival differences between groups were compared using the Log-rank test.

## 3 Results

### 3.1 Baseline characteristics

A total of 2,858 cases met the inclusion criteria, and 2,117 patients were ultimately included in the final cohort. The baseline characteristics of the training and validation sets are shown in **Table 1**. No significant differences were observed between the two groups across all baseline variables (p > 0.05), indicating a well-balanced data partition. In the overall cohort, the median age was 74 years, with the majority being male (79.5%) and white (88.6%). Small cell carcinoma was the most common histological subtype (76.9%). At diagnosis, a substantial proportion of patients presented with advanced disease, with 46.1% at stages III–IV and 80.4% at stages T2–T4, highlighting the aggressive nature of BNEC. With respect to treatment, 67.9% of patients underwent transurethral resection of bladder tumour (TURBT), 24.7% underwent radical cystectomy (RC), 61.3% received chemotherapy, and 23.5% received radiotherapy.

**Table 1:** Baseline clinical characteristics of BNEC patients.

Characteristic	Overall N = 2117	Training cohort N = 1482	Test cohort N = 635	p value
Age (%)				
≤74	1076 (50.8)	759 (51.2)	317 (49.9)	0.618
>74	1041 (49.2)	723 (48.8)	318 (50.1)	
Sex (%)				
Female	434 (20.5)	306 (20.6)	128 (20.2)	0.844
Male	1683 (79.5)	1176 (79.4)	507 (79.8)	
Race (%)				
White	1876 (88.6)	1314 (88.7)	562 (88.5)	0.963
Black	135 (6.4)	95 (6.4)	40 (6.3)	
Others	106 (5.0)	73 (4.9)	33 (5.2)	
Primary Site (%)				
Trigone	136 (6.4)	101 (6.8)	35 (5.4)	0.883
Lateral wall	449 (21.2)	307 (20.7)	142 (22.4)	
Anterior wall	114 (5.4)	78 (5.3)	36 (5.7)	
Posterior wall	219 (10.3)	158 (10.7)	61 (9.6)	
Bladder neck	96 (4.5)	67 (4.5)	29 (4.6)	
Overlapping lesion	367 (17.3)	256 (17.3)	111 (17.5)	
Bladder	736 (34.8)	515 (34.8)	221 (34.8)	
Histology (%)				
Large cell neuroendocrine carcinoma	61 (2.9)	36 (2.4)	25 (3.9)	0.278
Small cell carcinoma	1627 (76.9)	1152 (77.7)	475 (74.8)	
Oat cell carcinoma	3 (0.1)	2 (0.1)	1 (0.2)	
Combined small cell carcinoma	97 (4.6)	64 (4.3)	33 (5.2)	
Carcinoid tumor	8 (0.4)	4 (0.3)	4 (0.6)	
Neuroendocrine carcinoma	321 (15.2)	224 (15.1)	97 (15.3)	
AJCC Stage (%)				
0	8 (0.4)	6 (0.4)	2 (0.4)	0.896
I	308 (14.5)	216 (14.6)	92 (14.5)	
II	824 (38.9)	585 (39.5)	239 (37.6)	
III	288 (13.6)	202 (13.6)	86 (13.5)	
IV	689 (32.5)	473 (31.9)	216 (34.0)	
T Stage (%)				
T0/Ta/T1	415 (19.6)	293 (19.8)	122 (19.3)	0.983
T2	1140 (53.8)	795 (53.6)	345 (54.3)	
T3	302 (14.3)	213 (14.4)	89 (14.0)	
T4	260 (12.3)	181 (12.2)	79 (12.4)	
N Stage (%)				
N0	1651 (78.0)	1163 (78.5)	488 (76.9)	0.607
N1	187 (8.8)	133 (9.0)	54 (8.5)	
N2	213 (10.1)	141 (9.5)	72 (11.3)	
N3	66 (3.1)	45 (3.0)	21 (3.3)	
M Stage (%)				
M0	1660 (78.4)	1170 (78.9)	490 (77.2)	0.392
M1	457 (21.6)	312 (21.1)	145 (22.8)	
Surgery (%)				
No	136 (6.4)	93 (6.3)	43 (6.8)	0.381
TURBT	1437 (67.9)	998 (67.3)	439 (69.1)	
RC	522 (24.7)	378 (25.5)	144 (22.7)	
Others	22 (1.0)	13 (0.9)	9 (1.4)	
Chemotherapy (%)				
No/Unknown	820 (38.7)	578 (39.0)	242 (38.1)	0.736
Yes	1297 (61.3)	904 (61.0)	393 (61.9)	
Radiation (%)				
No/Unknown	1619 (76.5)	1136 (76.7)	483 (76.1)	0.812
Yes	498 (23.5)	346 (23.3)	152 (23.9)	

### 3.2 Analysis of independent prognostic factors

The results of univariate and multivariate Cox regression analyses are detailed in **Table 2**. In the univariate analysis, several variables were significantly associated with OS (p ≤ 0.05), including age >74 years, male sex, tumour origin at the bladder neck, histological type of carcinoid, T4 stage, lymph node metastasis, distant metastasis, surgery, radiotherapy, and chemotherapy. In the multivariate Cox regression model, age >74 years (HR = 1.51; 95% CI: 1.33–1.71; p<0.001), male sex (HR = 1.17; 95% CI: 1.01–1.36; p=0.038), advanced T stage (T4 vs. T0/Ta/T1; HR = 1.77; p<0.001), lymph node metastasis (N2 vs. N0; HR = 1.55; p<0.001), and distant metastasis (M1; HR = 1.99; p<0.001) were independently associated with poorer OS. Surgery (TURBT: HR = 0.75; p=0.017; RC: HR = 0.36; p<0.001), radiotherapy (HR = 0.79; p=0.002), and chemotherapy (HR = 0.43; p<0.001) were also independently associated with OS.

**Table 2:** Univariate and multivariate Cox regression analysis of overall survival in BNEC patients.

Characteristic	Univariate analysis	Multivariate analysis
Age		
≤74	Reference	
>74	1.80 (1.60–2.02, p<.001)	1.51 (1.33–1.71, p<.001)
Sex		
Female	Reference	
Male	1.20 (1.04–1.39, p=.014)	1.17 (1.01–1.36, p=.038)
Race		
White	Reference	
Black	1.24 (0.99–1.56, p=.059)	1.16 (0.92–1.45, p=.217)
Others	0.65 (0.49–0.88, p=.006)	0.65 (0.48–0.88, p=.005)
Primary Site		
Trigone	Reference	
Lateral wall	0.93 (0.72–1.21, p=.611)	1.04 (0.80-1.36, p=.759)
Anterior wall	0.93 (0.66–1.30, p=.657)	1.02 (0.72-1.43, p=.921)
Posterior wall	0.86 (0.64–1.14, p=.291)	0.87 (0.65–1.17, p=.365)
Bladder neck	1.41 (1.01–1.98, p=.044)	1.07 (0.76–1.51, p=.697)
Overlapping lesion	1.21 (0.93–1.58, p=.148)	1.24 (0.95–1.62, p=.115)
Bladder	1.18 (0.92–1.50, p=.194)	1.24 (0.96–1.59, p=.096)
Histological		
Large cell neuroendocrine carcinoma	Reference	
Small cell carcinoma	0.79 (0.56–1.11, p=.169)	0.91 (0.65–1.28, p=.597)
Oat cell carcinoma	0.35 (0.05–2.54, p=.298)	0.21 (0.03–1.53, p=.122)
Combined small cell carcinoma	0.84 (0.55–1.28, p=.409)	0.88 (0.57–1.35, p=.555)
Carcinoid tumor	0.12 (0.02–0.87, p=.036)	0.09 (0.01–0.63, p=.016)
Neuroendocrine carcinoma	0.94 (0.66–1.35, p=.747)	0.98 (0.68–1.41, p=.906)
AJCC stage		
0	Reference	
I	0.72 (0.30–1.76, p=.473)	
II	0.75 (0.31–1.81, p=.523)	
III	0.83 (0.34–2.03, p=.684)	
IV	1.72 (0.71–4.15, p=.230)	
T Stage		
T0/Ta/T1	Reference	
T2	1.02 (0.87–1.19, p=.847)	1.20 (1.03–1.41, p=.023)
T3	1.05 (0.86–1.29, p=.606)	1.63 (1.31–2.03, p<.001)
T4	1.91 (1.56–2.34, p<.001)	1.77 (1.42–2.21, p<.001)
N Stage		
N0	Reference	
N1	1.29 (1.06–1.57, p=.012)	1.38 (1.12–1.70, p=.002)
N2	1.89 (1.57–2.29, p<.001)	1.55 (1.26–1.92, p<.001)
N3	1.79 (1.29–2.46, p<.001)	1.19 (0.85-1.68, p=.311)
M Stage		
M0	Reference	
M1	2.60 (2.26–2.99, p<.001)	1.99 (1.70–2.34, p<.001)
Surgery		
No	Reference	
TURBT	0.50 (0.40–0.62, p<.001)	0.75 (0.59–0.95, p=.017)
RC	0.24 (0.19–0.31, p<.001)	0.36 (0.27–0.47, p<.001)
Other	0.39 (0.21–0.74, p=.004)	0.50 (0.26–0.95, p=.034)
Radiation		
No/Unknown	Reference	
Yes	0.77 (0.67–0.88, p<.001)	0.79 (0.68–0.92, p=.002)
Chemotherapy		
No/Unknown	Reference	
Yes	0.43 (0.38–0.48, p<.001)	0.43 (0.38–0.49, p<.001)

### 3.3 Establishment and evaluation of machine learning prediction models

On the basis of the independent prognostic factors identified from the multivariate Cox regression analysis, we developed eight machine learning models in the training set: GBM, RSF, Lasso-Cox, CoxBoost, SurvivalSVM, XGBoost, SuperPC, and plsRcox. The performance of the models was assessed in both the training and validation cohorts using the C-index and time-dependent ROC curves. In the training cohort, the time-dependent AUC values for predicting 1-, 3-, and 5-year survival for each model were as follows: GBM (1-year: 0.846, 95% CI: 0.827–0.865; 3-year: 0.825, 95% CI: 0.803–0.847; 5-year: 0.816, 95% CI: 0.792–0.839), RSF (1-year: 0.817, 95% CI: 0.796–0.839; 3-year: 0.815, 95% CI: 0.790–0.839; 5-year: 0.814, 95% CI: 0.789–0.840), LASSO-Cox (1-year: 0.808, 95% CI: 0.787–0.830; 3-year: 0.775, 95% CI: 0.751–0.800; 5-year: 0.764, 95% CI: 0.738–0.790), XGBoost (1-year: 0.794, 95% CI: 0.772–0.816; 3-year: 0.766, 95% CI: 0.742–0.791; 5-year: 0.753, 95% CI: 0.727–0.778), Coxboost (1-year: 0.814, 95% CI: 0.793–0.836; 3-year: 0.781, 95% CI: 0.757–0.805; 5-year: 0.768, 95% CI: 0.742–0.794), and plsRcox (1-year: 0.815, 95% CI: 0.794–0.836; 3-year: 0.783, 95% CI: 0.759–0.807; 5-year: 0.770, 95% CI: 0.744–0.796); SurvivalSVM and SuperPC performed poorly, with AUC values significantly lower than other models at all time points. In the validation cohort, the AUC values for each model were as follows: GBM (1-year: 0.737, 95% CI: 0.698–0.775; 3-year: 0.730, 95% CI: 0.687–0.773; 5-year: 0.742, 95% CI: 0.697–0.787), LASSO-Cox (1-year: 0.722, 95% CI: 0.683–0.761; 3-year: 0.719, 95% CI: 0.677–0.762; 5-year: 0.730, 95% CI: 0.685–0.775), plsRcox (1-year: 0.723, 95% CI: 0.684–0.762; 3-year: 0.722, 95% CI: 0.679–0.765; 5-year: 0.732, 95% CI: 0.687–0.777), XGBoost (1-year: 0.717, 95% CI: 0.678–0.756; 3-year: 0.704, 95% CI: 0.661–0.747; 5-year: 0.718, 95% CI: 0.672–0.763), CoxBoost (1-year: 0.722, 95% CI: 0.682–0.761; 3-year: 0.720, 95% CI: 0.677–0.763; 5-year: 0.730, 95% CI: 0.685–0.775), RSF (1-year: 0.691, 95% CI: 0.650–0.732; 3-year: 0.677, 95% CI: 0.627–0.728; 5-year: 0.683, 95% CI: 0.629–0.737) (**Figure 2**).

**Figure 2:**
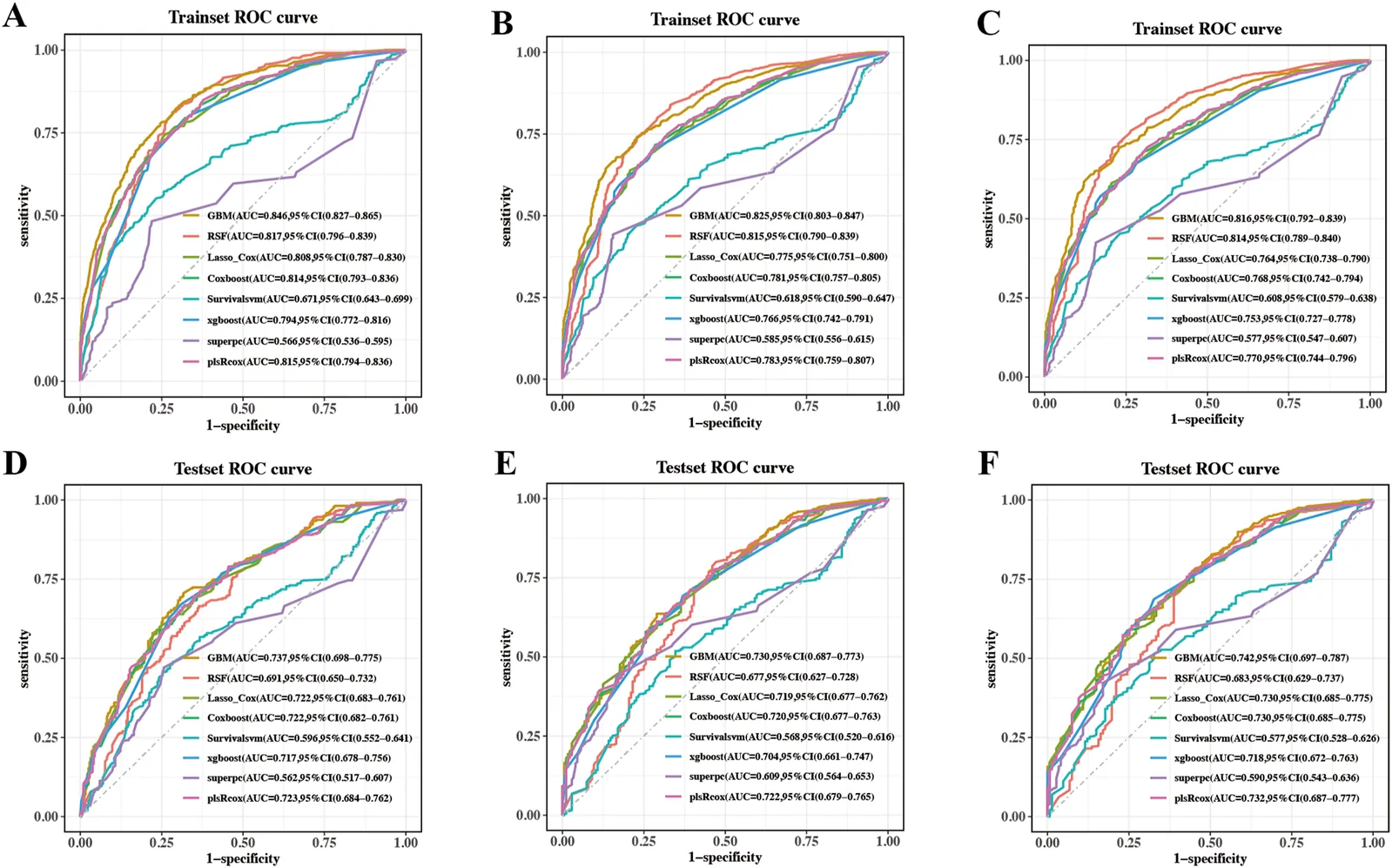
Receiver operating characteristic (ROC) curves. **(A–C)** ROC curves for the 1-, 3-, and 5-year predictions in the training set, respectively. **(D–F)** ROC curves for the 1-, 3-, and 5-year predictions in the validation set, respectively.

Consistent with the time-dependent AUC results, the concordance index (C-index) analysis showed that the GBM model had the highest C-index values among the evaluated models in both the training and validation cohorts (**Figure 3**). Compared with the other models, the GBM model consistently showed higher C-index values across the evaluated time points, with a relatively stable trend over time. Notably, the C-index of the GBM model remained above 0.69 at the 60-month time point, indicating sustained discrimination during long-term follow-up. The similar curve patterns between the training and validation cohorts further supported the consistency of the model performance. To further compare the machine learning model with a conventional prognostic approach, we constructed a TNM-based model using the same validation cohort. Time-dependent ROC analysis showed that the TNM-based model had lower AUC values than the GBM model at 1, 3, and 5 years (**Figure 4**). Taken together, the GBM model showed the highest observed C-index and time-dependent AUC values among the evaluated models and relatively stable performance across the evaluated time points. Compared with the conventional TNM-based model, the GBM model also showed higher time-dependent AUC values at 1, 3, and 5 years. Based on these findings, the GBM model was selected for subsequent survival prediction in patients with BNEC.

**Figure 3:**
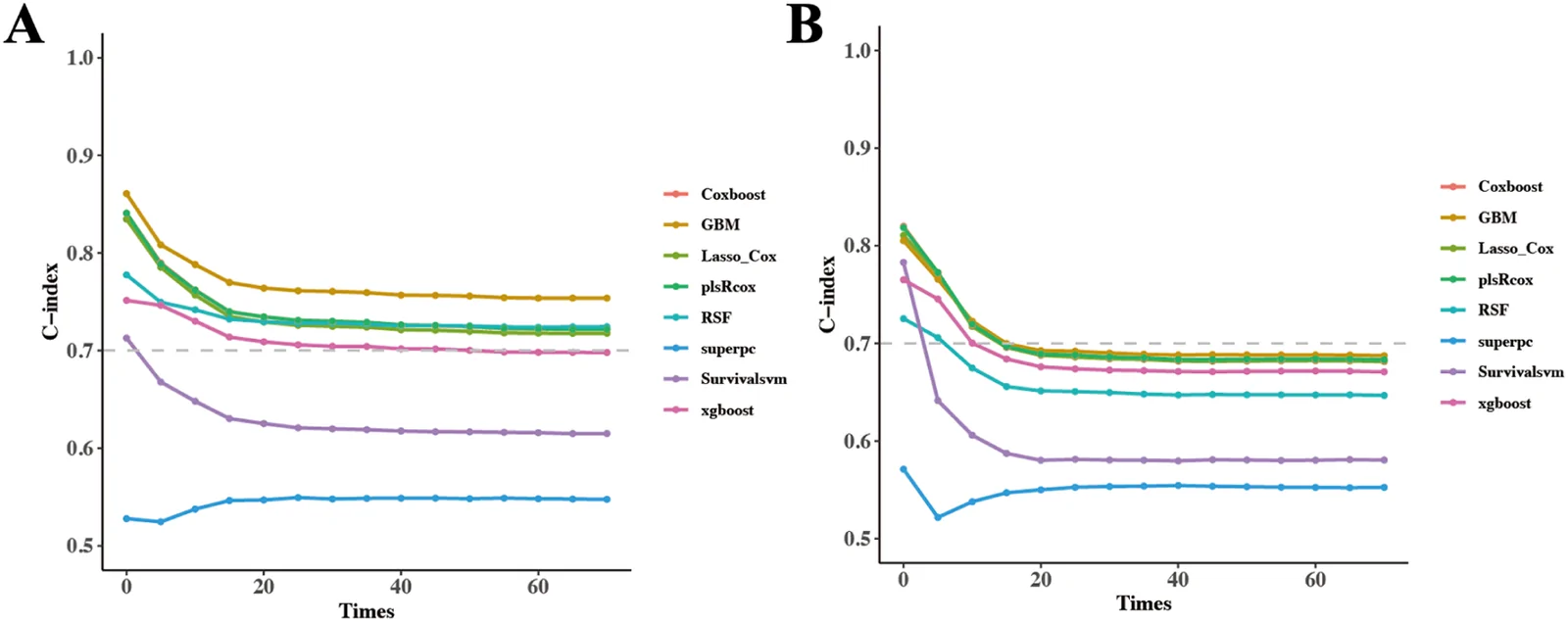
Comparison of concordance index (C-index) values across machine learning models. **(A)** Comparison of C-index values among different machine learning models in the training set. **(B)** Comparison of C-index values among different machine learning models in the validation set.

**Figure 4:**
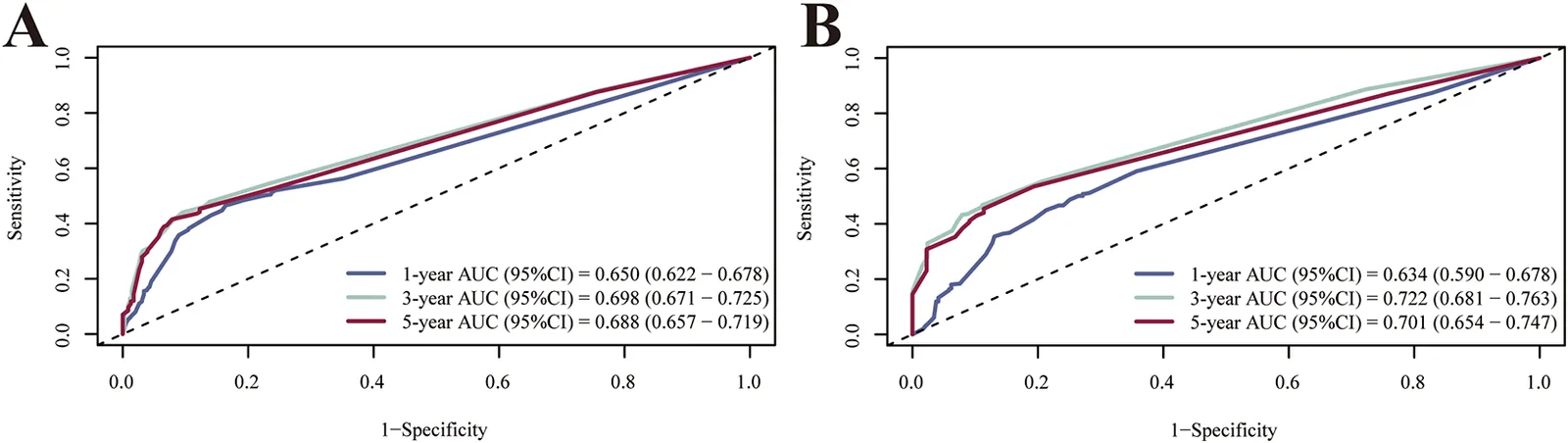
Receiver operating characteristic (ROC) curves for TNM staging. **(A)** ROC curve in the training set. **(B)** ROC curve in the validation set.

### 3.4 Performance validation of the GBM model

To further assess the clinical applicability and robustness of the GBM model, we performed calibration curve analysis and decision curve analysis (DCA) in both the training and validation cohorts. At all assessment time points (1 year, 3 years, and 5 years), the data points representing the GBM predictions clustered closely around the reference line (diagonal), indicating excellent calibration with highly consistent agreement between the predicted and observed values. With respect to the 1-year survival predictions, the calibration curve nearly coincided with the diagonal line, particularly in the medium-to-high probability range, indicating good agreement between the predicted and observed probabilities. As the prediction horizon extends to 5 years, the observed values slightly exceed the predicted values in the low-probability range. This suggests that the model may exhibit a slight systematic underestimation in this interval, although the deviation remains within clinically acceptable limits (**Figure 5**). Calibration curve analysis demonstrated that the GBM model exhibited excellent accuracy across different time points, yielding highly credible survival probability outputs. Overall, the calibration results confirm that the GBM model delivers highly reliable and clinically credible survival probability estimates across all evaluated time points. However, accurate prediction alone does not guarantee clinical usefulness. Therefore, to determine whether these predictions can translate into tangible patient benefit in real-world decision-making, we subsequently conducted decision curve analysis (DCA).

**Figure 5:**
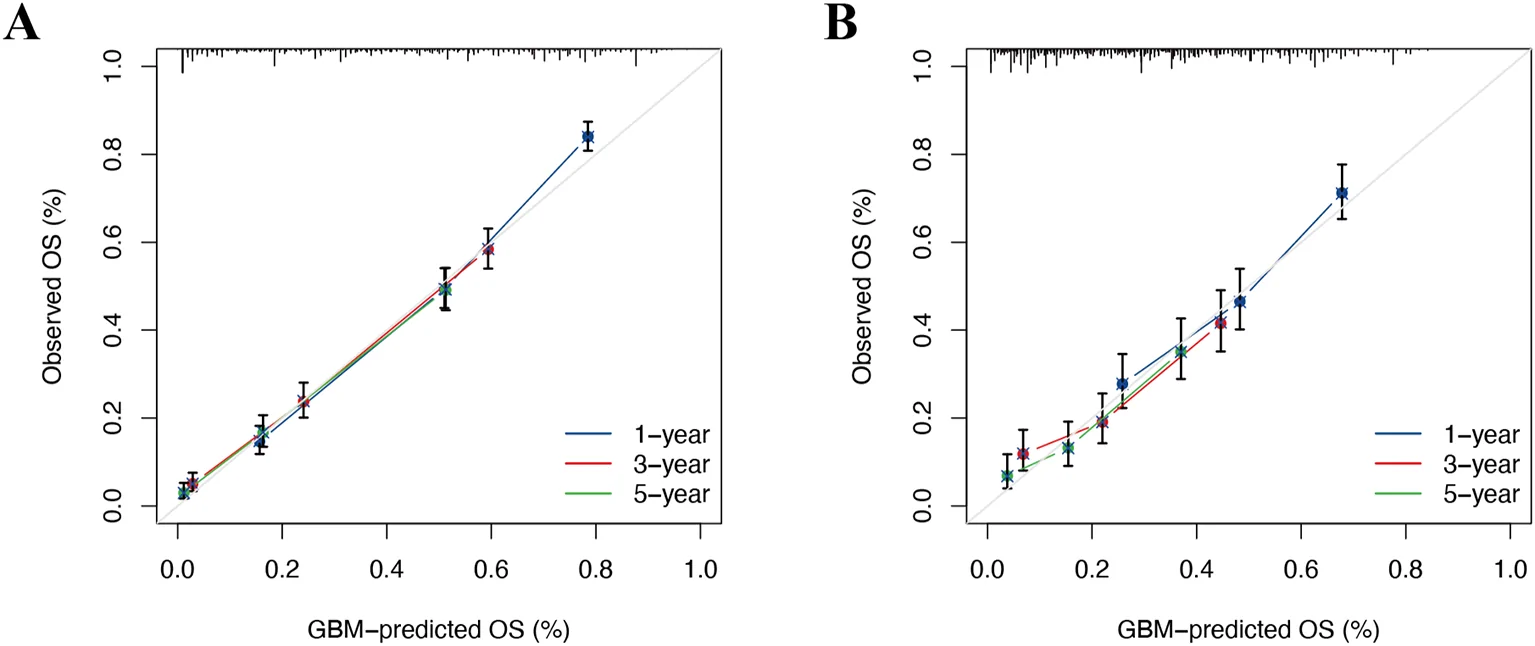
Calibration curves for the prediction model. **(A)** Calibration curve in the training set. **(B)** Calibration curve in the validation set.

As shown, the net benefit curve of the GBM model significantly exceeds both reference lines across a broad range of threshold probabilities. Specifically, in the training set, its clinically effective range covers 0.05–0.75 for 1-year predictions, 0.10–0.70 for 3-year predictions, and 0.15–0.65 for 5-year predictions (**Figures 6A–C**). Although the range narrows slightly in the validation set, it remains within commonly used clinical decision thresholds, indicating stable practicality across different prediction time points. The net benefit of the GBM model peaks within the clinically most significant moderate-risk threshold. For instance, in the 1-year prediction, the net benefit value is approximately 0.35 at a threshold probability of 0.3 (**Figures 6D–F**). These findings suggest that the GBM model may provide additional net benefit for prognostic risk stratification across a range of threshold probabilities.

**Figure 6:**
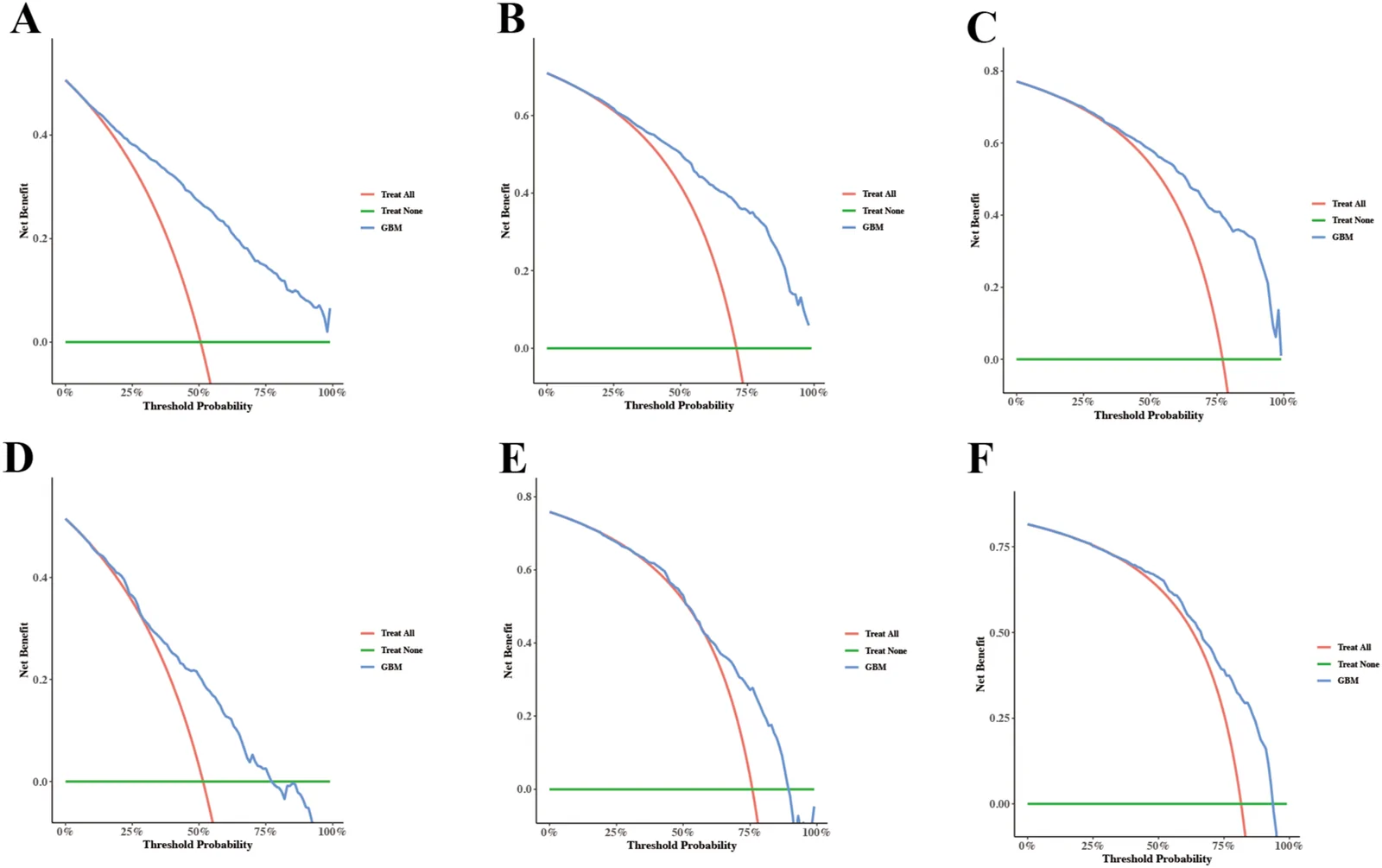
Decision curve analysis (DCA). **(A–C)** DCA curves for the 1-, 3-, and 5-year predictions in the training set, respectively. **(D–F)** DCA curves for the 1-, 3-, and 5-year predictions in the validation set, respectively.

DCA provides compelling evidence that the GBM model is not merely an “ivory tower” tool with high statistical metrics but also a reliable assistant offering substantive support in real-world clinical decision-making. It effectively differentiates patient risk, providing useful information for prognostic assessment and risk stratification across different threshold probabilities.

### 3.5 Risk stratification ability of the GBM model

On the basis of risk scores calculated by the GBM model, patients were categorized into high-risk and low-risk groups. Kaplan–Meier analysis revealed that in the training set, survival curves for both groups differed significantly from the initial follow-up and maintained a substantial difference throughout. Patients in the low-risk group demonstrated favourable long-term survival potential, with significantly prolonged median survival. Statistical testing (log-rank test) revealed extremely high statistical significance between the groups (p < 0.001) (**Figure 7**). These findings demonstrate that the GBM model can identify patient subgroups with different prognoses, providing useful information for individualized prognostic assessment and risk stratification.

**Figure 7:**
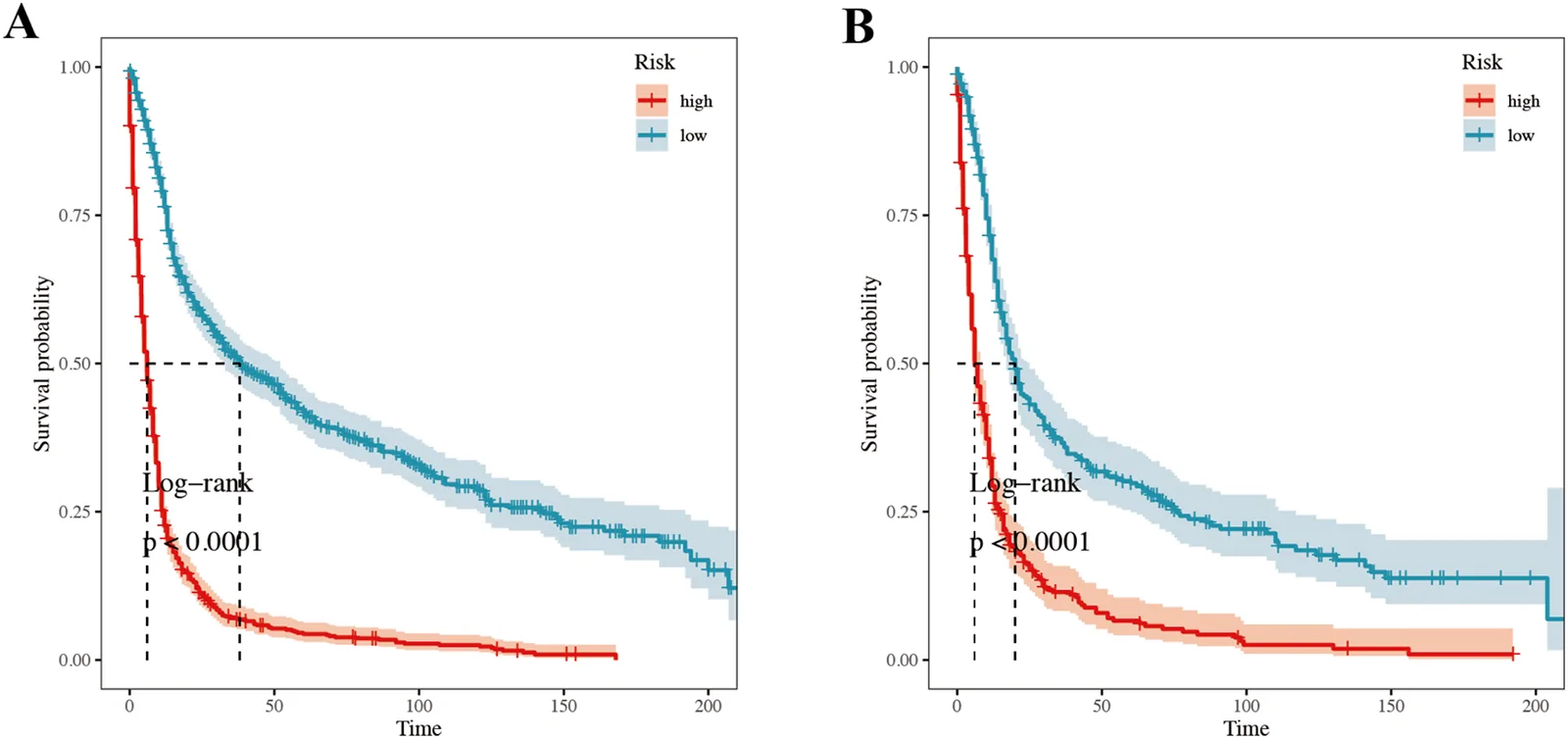
Kaplan–Meier (K–M) survival analysis based on the GBM model. **(A)** Survival analysis in the training set. **(B)** Survival analysis in the validation set.

## 4 Discussion

In recent years, the application value of machine learning in medical prognosis prediction has become increasingly prominent. Its advantage lies in the ability to process high-dimensional, nonlinear complex data and automatically identify potential interactions between variables, thereby surpassing the predictive efficacy of traditional statistical models. This advantage has been preliminarily validated in studies of various tumours. This trend is evident across multiple tumour types. For instance, in hepatocellular carcinoma research, a GBM model constructed using contrast-enhanced ultrasound (CEUS) imaging features and LI-RADS classification effectively predicted early postoperative recurrence (C-index of 0.802) (7); in breast cancer patients with brain metastases, algorithms such as XGBoost, which integrates clinical and molecular features enabled high-precision prediction of survival outcomes. Furthermore, ML has demonstrated outstanding performance in areas such as metabolic fragility analysis for osteosarcoma, diagnostic and prognostic models for gastric cancer, and radiomics-based recurrence prediction for endometrial cancer (7, 14, 15). Despite the promising prospects of machine learning methods, their systematic application in BNEC—a rare and highly aggressive malignancy—remains unexplored. Compared with traditional prognostic factors (e.g., TNM staging), machine learning models can comprehensively incorporate key information such as demographic characteristics and treatment patterns, offering the potential for more precise individualized prognosis assessment. In this context, for the first time, this study utilized large-scale population data from the US SEER database to systematically develop and validate the performance of eight machine learning algorithms in predicting BNEC survival.

The independent prognostic factors identified in this study were broadly consistent with those reported in previous studies (10, 11). Advanced age, male sex, and higher TNM stage were associated with poorer overall survival, while surgery, chemotherapy, and radiotherapy were also identified as prognostic variables in our cohort. Carcinoid tumor was also associated with better overall survival in the multivariable analysis (HR = 0.09, P = 0.016); however, given its limited sample size, this finding should be interpreted cautiously as an exploratory association. Further studies with larger multicentre cohorts are needed to evaluate prognostic heterogeneity across different histological subtypes of BNEC.Previous studies have incorporated clinicopathological characteristics into prognostic nomograms for patients with BNEC (16). In our cohort, the GBM model demonstrated better discrimination than TNM staging alone, suggesting that integrating multiple clinical and pathological characteristics may provide additional prognostic information beyond conventional staging. Compared with conventional prognostic approaches, the machine learning framework used in this study integrates multiple variables to generate individualized estimates of survival probability.

Compared with traditional Cox regression models or models in recent publications (10, 11) the GBM model in this study demonstrated greater predictive accuracy (e.g., the C-index of the prediction model developed by Li et al) developed predictive models with C-index values ranging from 0.702 to 0.724. This advantage stems from the machine learning algorithm’s robust ability to handle complex nonlinear relationships and interactions among variables. Furthermore, we demonstrated the clinical utility of the model through decision curve analysis (DCA), an essential component often overlooked in many prognostic studies (17). DCA addresses the critical question:”Does using this model yield clinical benefit?” Similar challenges have been reported in machine learning-based prediction studies involving relatively small or imbalanced clinical datasets (16, 18). Given the rarity of BNEC, the moderate discrimination observed in our study should be interpreted in the context of limited sample size and outcome distribution. Direct comparisons across different diseases and datasets should be made cautiously because differences in patient characteristics, sample size, outcome definitions, and follow-up may substantially affect model performance.

This study has several limitations. First, its retrospective design and the inherent characteristics of the SEER database may introduce selection and information biases. Second, the SEER database lacks several potentially relevant clinical and pathological variables, including specific chemotherapy regimens, radiotherapy doses, detailed surgical information (e.g., margin status), and pathological markers such as the Ki-67 proliferation index. The absence of these variables may limit the comprehensiveness of the prognostic model. Third, although the model was internally evaluated using a 7:3 training-validation split and bootstrap resampling with optimism correction, independent external validation was not performed. In addition, the associations between treatment-related variables and survival may be affected by confounding by indication and immortal time bias and therefore should not be interpreted as causal treatment effects (19, 20). Given the rarity of BNEC, an independent cohort with a sufficiently large sample size and comparable clinical variables was not available for the present study. Therefore, the generalizability and transportability of the model to other populations remain uncertain and should be further evaluated in independent multicentre cohorts (12, 13).

In the future, we will focus on collecting multicentre external data with richer features for validation. Concurrently, the integration of multimodal data such as radiomics and genomics data (e.g., TP53 and RB1 mutations) represents a crucial direction for further enhancing the model’s predictive accuracy and achieving true precision medicine.In the future, we will focus on collecting multicentre external data with richer features for validation. Concurrently, the integration of multimodal data such as radiomics and genomics data (e.g., TP53 and RB1 mutations) represents a crucial direction for further enhancing the model’s predictive accuracy and achieving true precision medicine.

## 5 Conclusion

In this study, a survival prediction model for BNEC patients was constructed and validated using a machine learning GBM algorithm. The model demonstrated good predictive performance and effectively distinguished patients with different levels of prognostic risk. It may provide a quantitative tool for individualized prognostic assessment and risk stratification and may also provide useful information for patient counselling.

## Data Availability

The raw data supporting the conclusions of this article will be made available by the authors, without undue reservation.
